# Primary Hepatic Non-Hodgkin’s Lymphoma: An Enigma Beyond the Liver, a Case Report

**DOI:** 10.14740/wjon900w

**Published:** 2015-04-12

**Authors:** Shalini Thapar Laroia, Archana Rastogi, Dipanjan Panda, Shiv Kumar Sarin

**Affiliations:** a Department of Radiology, Institute of Liver and Biliary Sciences, Sector D-1, Vasant Kunj, New Delhi 110070, India; b Department of Hepatopathology, Institute of Liver and Biliary Sciences, Sector D-1, Vasant Kunj, New Delhi 110070, India; c Department of Oncology, Institute of Liver and Biliary Sciences, Sector D-1, Vasant Kunj, New Delhi 110070, India; d Department of Hepatology, Institute of Liver and Biliary Sciences, Sector D-1, Vasant Kunj, New Delhi 110070, India

**Keywords:** Primary hepatic lymphoma, NHL, Diffuse large cell B cell lymphoma, Renal involvement, MRI, Localized spread

## Abstract

We have discussed a unique presentation of primary diffuse large cell B-cell non-Hodgkin (DLBC NHL) hepatic lymphoma involving the porta hepatis and biliary confluence causing obstructive jaundice with contiguous soft tissue involvement of the right lobe of liver extending up to the right renal cortex. This appears to be the only case in literature where primary hepatic lymphoma has shown contiguous localized intra- and extrahepatic tumor infiltration. A 67-year-old gentleman presented with history of significant loss of appetite and weight in 2 months with associated progressive painless cholestatic jaundice. Physical evaluation revealed normal vitals with pallor, deep icterus, scratch marks over the abdomen, generalized muscle wasting, grade II clubbing and a palpable non-tender liver with a globular, firm mass beneath the liver. He had a total serum bilirubin of 15.9 mg/dL and direct bilirubin of 9.24 mg/dL. His liver enzymes were moderately elevated with raised serum creatinine and dyselectrolytemia. Serology for enterohepatic viruses was negative. Contrast-enhanced magnetic resonance imaging (CEMRI) showed poorly enhancing multiple soft tissue masses in both lobes of liver with the largest mass involving, biliary confluence and porta hepatis causing right bile duct and portal vein encasement. The mass occupied the posterior right lobe and extended to the inferior surface of liver with contiguous invasion of the right renal upper pole cortex. The mass was associated with a retracted liver capsule in the involved segments and delayed enhancement, mimicking a cholangiocarcinoma. Tissue biopsy revealed hepatic DLBC type NHL and patient was subsequently treated with a CHOP-R (cyclophosphamide-doxorubicin-vincristine-prednisolone/rituximab) regimen, on which he has shown non-progressive disease at 1-year follow-up. DLBC NHL of the liver is a very rare tumor with propensity for isolated involvement of the liver and minimal extrahepatic spread. This case shows many interesting features such as obstructive jaundice for 2 months, porta hepatis involvement and tumor infiltration up to the right renal parenchyma. We have illustrated various imaging findings which should be considered when evaluating such a lesion to help differentiate it from cholangiocarcinoma. The literature is extensively reviewed. The case demonstrates relevant diagnostic parameters for physicians, radiologists and oncologists who are likely to encounter patients with tumor-induced obstructive jaundice in their daily practice.

## Introduction

Lymphoma is a systemic lymphoproliferative malignancy and is classified into the Hodgkin and non-Hodgkin lymphoma (NHL) subtypes. NHL originates from nodal and extra nodal sites. Primary hepatic lymphoma (PHL) is a very rare subgroup of extra-nodal NHL because of a natural lack of abundant lymphoid tissue in the liver. It is seen in less than 1% of NHL [1, 2]. PHL was first described by Ata and Kamel in 1965 [3].

The diagnostic criterion for PHL is a lesion confined to the liver without spleen, bone marrow or hematological involvement and lack of superficial lymphadenopathy at the time of presentation as well as up to the next 6 months [4]. The criterion also includes lesions which are primarily involving the liver and have minor involvement of other organs or small abdominal lymph nodes [5].

Due to the stringent and well-defined diagnostic guidelines of this group of lymphomas, only few hundred isolated case reports have been described in literature so far. Most of these tumors present as insidious, asymptomatic lesions which are rarely suspected at the time of evaluation and are a histological surprise [6]. The spectrum of clinical symptoms varies from asymptomatic patients at one extreme and presents with fulminant hepatic failure at the other extreme. Obstructive jaundice is an uncommon and late feature of hepatic NHL. It may be seen as a presenting symptom in less than 2% of patients with NHL [7]. The different patterns of the tumor on imaging include solitary, multiple nodules and diffuse infiltrative mass lesion seen in equally prevalent proportions [8]. The patient in our study presented with painless obstructive jaundice for a short duration of 8 weeks and showed multiple lesions in the liver with a predominant infiltrative mass involving the porta hepatis, biliary confluence, right posterior lobe of liver and contiguous soft tissue involvement of the right kidney, which are all extremely uncommon features of PHL in the NHL group.

The etio-pathogenesis of PHL is unclear; however, few associated factors have been suggested. These include: prior infection with hepatitis B virus (HBV), hepatitis C virus (HCV), Epstein-Barr virus (EBV), cirrhosis due to primary biliary or secondary to viral infections, auto-immune diseases and immunosuppressive therapy [8, 9]. It has also been hypothesized that PHL is actually an amalgam of various histologies such as high-grade lymphoblastic and Burkett’s type, hepato splenic T-cell, follicular, mucosa-associated lymphoid tissue type (MALT), anaplastic large-cell, T-cell-rich or B-cell rich lymphomas. The commonest is the DLBCL variety. Main patterns of liver involvement on histopathology are: portal infiltrates nodular growth, sinusoidal growth, loose infiltration and dense infiltration. They are seen as sheets of loosely cohesive, large atypical lymphocytes with moderate amount of cytoplasm, enlarged round-irregular vesicular nuclei with prominent nucleoli and frequent mitoses.

Although there are few case reports of surgically treated solitary focal liver lesions in PHL, the universally accepted non-surgical treatment consists of cyclophosphamide, doxorubicin, vincristine, prednisone and rituximab (CHOP-R) regimen, which is the mainstay of therapy for PHL patients.

We present a unique case of NHL with obstructive jaundice involving the liver as the primary site. We believe that the soft tissue extension of the tumor to the right kidney along with the other imaging and clinical features makes this a rare case report which is worthy of attention to all physicians and clinicians who observe similar cases in their daily practice.

## Case Report

A 67-year-old gentleman presented with history of significant loss of appetite and weight (approximately 42 pounds) in 2 months with associated progressive painless cholestatic jaundice. He also complained of extreme generalized weakness and fatigue. The patient denied any febrile episodes or bleeding manifestations during this period. There was no history of abdominal pain, distension or fullness. No symptoms of altered sensorium leg and face swelling or decreased urine output were seen. Patient denied chronic, addictive substance abuse or use of any indigenous medication during and prior to his illness period. The patient had never undergone surgery or blood transfusions in the past. Physical evaluation revealed the patient was conscious, oriented and afebrile with normal vitals. Pallor was evident and the patient was deeply icteric with evidence of scratch marks over the abdomen. There was generalized muscle wasting and loss of subcutaneous fat over the face and hollowing of the temporal regions. There was presence of grade II clubbing. No evidence of cyanosis or peripheral edema was seen. No peripheral, mediastinal or abdominal adenopathy was found. The abdomen was non-distended without visible venous prominences or visible pulsations. Palpation revealed a firm, rounded, smooth non-tender liver palpable up to 6 cm below the right costal margin. A large globular non-tender firm mass of about 8 cm was palpable beneath the liver. There was no splenomegaly or free fluid. The rest of the systemic examination was normal and non-contributory. His laboratory investigations are shown in Table 1.

**Table 1:** Investigation Chart

Investigation	Results
Hemoglobin	9.3 g/dL (normal: < 1.0)
Total leukocyte count	15,900/mm^3^ (normal: 4 - 11 × 10^9^)
Platelet count	3.16 lakh (normal: 1.5 - 4 lakh)
Prothrombin time/INR	14.7/1.24 corrected, received vitamin K
Total serum bilirubin	15.9 mg/dL (normal: 0.3 - 1.2 mg/dL)
Direct bilirubin	9.24 mg/dL (normal: 0 - 0.2 mg/dL)
Indirect bilirubin	6.66 mg/dL (normal: 0.2 - 0.8 mg/dL)
AST	174 IU/L (normal: 5 - 40 IU/L)
ALT	121 IU/L (normal: 10 - 40 IU/L)
SAP	1,106 IU/L (normal: 32 - 92 IU/L)
GGTP	474 IU/L (normal: 7 - 64 IU/L)
Alb/Glob	0.5 (normal: 1.5 - 2.5)
AFP	4.03 ng/mL (normal: 0 - 8.5 ng/mL)
CEA	5.68 U/mL (normal: 0 - 37 U/mL)
Serum CA 19-9	428.3 U/mL (normal: 0 - 37 U/mL)
Serum creatinine	0.91 mg/dL (normal: 0.2 - 1 mg/dL)
Serum LDH	889 IU/dL (normal: 265 - 400 IU/L)

**Figure 1:**
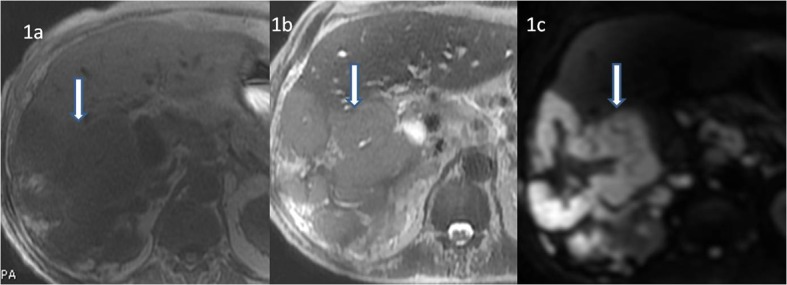
Non-contrast MRI sequences of the liver showing lesions in both lobes. (a) Lesions appear hypointense (white bold arrow) on T1-weighted sequence of non-contrast MRI. (b) Lesions appear hyperintense on T2-weighted sequence (bold white arrow). (c) Lesions show restriction (bold white arrow) on diffusion weighted images.

**Figure 2:**
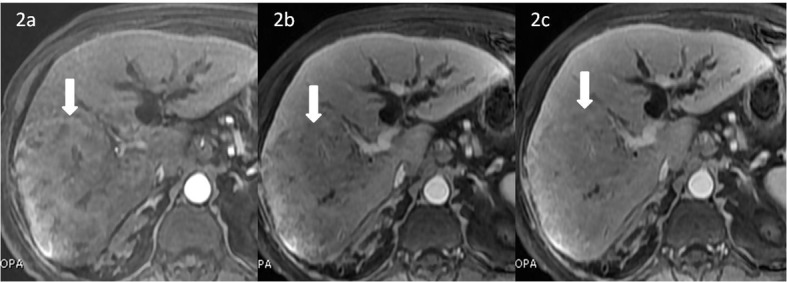
Dynamic contrast-enhanced MRI (CEMRI) T1-weighted fat suppressed sequence using hepatocyte specific contrast gadobenate dimeglumine (Gd BOPTA) showing lesion enhancement pattern. (a) Lesion in the right lobe (white bold arrow pointing down) shows mild enhancement in the hepatic arterial phase (HAP). (b) Lesion appears relatively hypointense with better visualization of the lesion (white bold arrow pointing down) on the portal venous phase (PVP). (c) Lesion appears iso-hypointense compared to rest of the liver parenchyma (white bold arrow pointing down) on the equilibrium phase.

**Figure 3:**
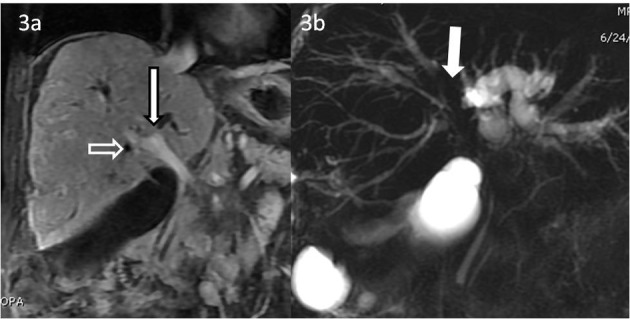
CEMRI T1-weighted fat suppressed sequence performed with gadolinium BOPTA showing lesion extent on the coronal sequences. (a) Coronal image of the dynamic CEMRI T1-weighted fat suppressed sequence performed with Gd BOPTA in the equilibrium phase showing lesion extension to the porta hepatis (bold white arrow with black margins) and encasement of the right portal vein (white outlined arrow). (b) Coronal 3D MRCP shows non-visualized primary biliary confluence with resultant moderate left lobe and mild right lobar ductal dilatation (bold white arrow pointing at the confluence).

**Figure 4:**
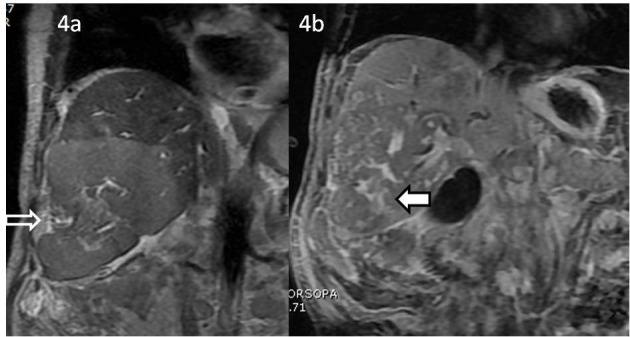
MRI coronal FIESTA (a) and coronal post contrast T1-weighted fat suppressed images after 1 h (b). (a) MRI coronal FIESTA sequence shows striking areas of capsular retraction (white arrow) along the right lobe of liver abutting the mass. (b) MRI post contrast (Gd BOPTA) coronal T1-weighted fat suppressed sequence images acquired after 1 h delay reveal delayed enhancement within few central areas of the mass (bold white arrow with black margins).

**Figure 5:**
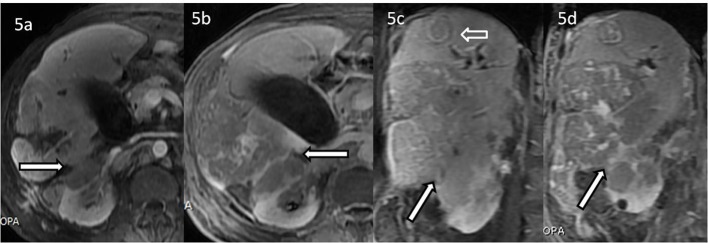
Post contrast MRI sequences describe the loss of fat planes between the tumour and right kidney. (a) Axial late arterial phase post contrast MRI T1-weighted fat suppressed sequence showing the loss of fat planes between right renal upper cortex and the liver mass in the right lobe of liver showing mild enhancement but relatively hypointense to the liver (bold white arrow with black outline). (b) Axial portal venous phase post contrast MRI T1-weighted fat suppressed sequence showing the loss of fat planes between right renal upper cortex and the liver mass in the right lobe of liver (bold white arrow with black outline). (c) Coronal equilibrium phase post contrast MRI T1-weighted fat suppressed sequence showing the loss of fat planes between right renal upper cortex and the liver mass in the right lobe of liver appearing enhanced, however hypointense compared to the liver parenchyma (bold white arrow with black outline). Note is made of another target like lesion in the liver (white arrow). (d) Coronal delayed (1 h) phase post contrast MRI T1-weighted fat suppressed sequence showing the loss of fat planes between right renal upper cortex and the liver mass in the right lobe of liver showing areas of enhancement, however appearing hypointense compared to the liver parenchyma (bold white arrow with black outline).

**Figure 6:**
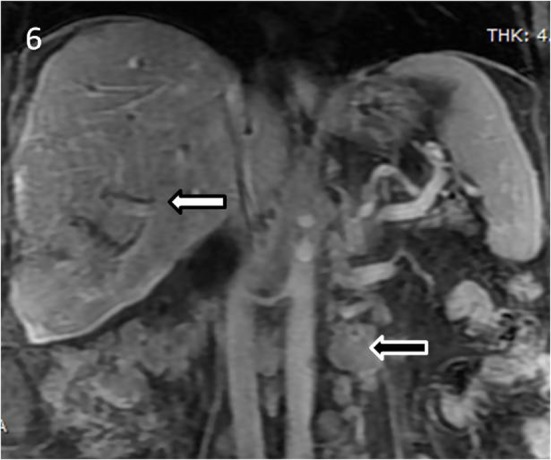
Coronal post contrast MRI T1-weighted fat suppressed sequence showing few prominent (approximately 1 cm in diameter), para-caval and para-aortic lymph nodes were seen in the retroperitoneum.

**Figure 7:**
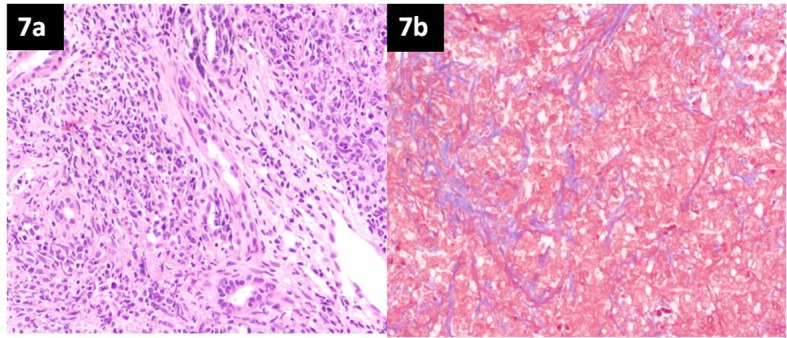
Hematoxylin and eosin (H&E) staining of liver tissue. (a) Liver biopsy showing portal and parenchymal diffuse infiltration by monomorphic large atypical lymphoid cells (H&E), magnification × 40. (b) Sheets of singly lying atypical large lymphoid cells with intervening sclerosis (blue) on Masson’s trichrome stain, magnification × 40.

**Figure 8:**
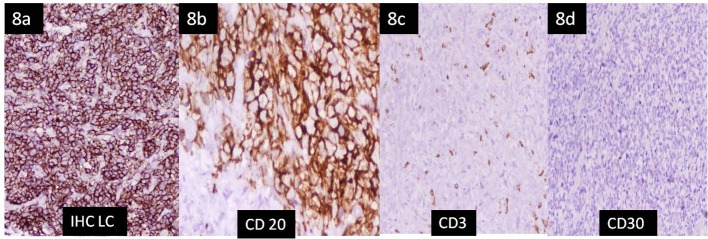
Immunohistochemistry of the liver tissue. (a, b) Strong diffuse membranous staining of atypical cells with leukocyte common antigen (magnification × 100) and CD20 (B-cell marker) (magnification × 200) respectively. (c, d) Tumor cells are negative for CD3 (T-cell marker) and CD30 (× 40).

**Figure 9:**
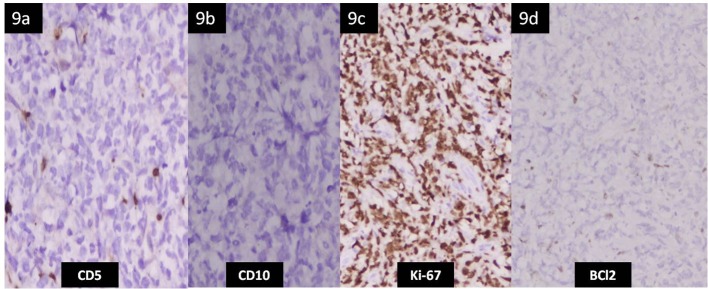
Immunohistochemistry of the liver tissue. (a, b, d) Atypical lymphoid cells are negative for CD5, CD10 and Bcl2 (× 100). (c) Ki67 immunohistochemistry shows positive nuclear staining in > 80% of the tumor cells.

Serology for hepatitis A, E, B, and C viruses as well as for HIV was negative. Tumor markers such as carcinoembryonic antigen (CEA) and alpha-fetoprotein (AFP) were normal. Serum CA19-9 was elevated. Contrast-enhanced magnetic resonance imaging (CEMRI) using hepatocyte specific contrast gadobenate dimeglumine (gadolinium BOPTA) was performed at our institute which revealed the presence of multiple T1-hypointense and T2-hyperintense intrahepatic (entire right lobe and segment-IV of the left lobe) lesions showing restriction on diffusion weighted images (Fig. 1). On contrast administration, these lesions showed mild, predominantly peripheral, arterial enhancement and appeared relatively hypointense on venous and delayed phase (Fig. 2). The mass was seen encasing and attenuating the right portal vein and its branches (Fig. 3a). The largest lesion in the right lobe was seen extending to the hilum, involving primary and right secondary biliary confluence with resultant bilobar intrahepatic biliary radicles (IHBR) dilatation (Fig. 3b). The gall bladder was over-distended. Striking areas of capsular retraction were noted along the right lobe mass (Fig. 4a). Delayed enhancement within few central areas of the mass was present on the 1 h post hepatocyte specific contrast acquisitions (Fig. 4b). The mass was seen to occupy almost the entire right lobe and extend to the inferior surface of liver with contiguous invasion of the right renal upper pole cortex (Fig. 5). Few prominent sub-centimeter para-caval and para-aortic lymph nodes were seen in the retroperitoneum (Fig. 6). The mass was associated with a retracted liver capsule in the involved segments and delayed enhancement, mimicking a cholangiocarcinoma. Tissue biopsy revealed hepatic DLBC type NHL (Fig. 7-9). Bone marrow aspirate and biopsy were negative for malignancy. Various other differential diagnoses (as listed below) were excluded by doing an extensive panel of immunohistochemical markers, demonstrated in Table 2.

**Table 2:** Immunohistochemistry Results

Positive	Negative
CD45 (leukocyte common antigen)	CD3 (T lineage marker)
CD20 (B lineage marker)	HepPar-1, glypican-3 (hepatocellular carcinoma)
CD10	Vimentin(mesenchymal cell marker)
CD5	Synaptophysin chromogranin (neuroendocrine markers)
Bcl2	CK7, CK19 (markers for mmetastatic carcinoma and cholangiocarcinoma)
Ki67	Pancytokeratin

Differential diagnosis on imaging as well as on pathology which was ruled out at tissue biopsy is as follows: 1) hepatocellular carcinoma; 2) cholangiocarcinoma; 3) metastatic carcinoma; 4) metastatic neuroendocrine carcinoma; 5) malignant lymphoma (T-cell rich B-cell lymphoma, marginal zone B-cell lymphoma, anaplastic large cell lymphoma, sinusoidal T-cell lymphoma, and Hodgkin’s lymphoma).

He was subsequently treated with a CHOP-R regimen, on which he is doing well at 1-year follow-up.

## Discussion

PHL is defined as tumor confined to the liver with no evidence of lymphomatous or hematological involvement of other organs in the body. It is rare to find NHL presenting with obstructive jaundice. The patient in our study is of interest because of the rarity of combination of PHL presenting as malignant biliary obstruction. This particular tumor combined multiple interesting features such as multiple hepatic lesions, involvement of porta hepatis, portal vein encasement and biliary obstruction. However the most noteworthy feature that was confounding in its diagnosis was, the fact that the tumor extended to the right renal parenchyma directly from the right lobe of liver crossing the hepatic capsule and Morrison’s pouch fat. This is probably the first case in literature which exhibited such findings. Literature review of published case reports of DLBCL of the liver reveals that presentation of these tumors is an amalgam of various entities [10]. The median age of presentation of PHL has been documented as 50 years although it is seen in the age group of 30 - 90 years [11]. Most of these lesions show indeterminate imaging findings and non-specific clinical symptoms [12].

Lymphomatous liver lesions may be incidentally diagnosed on imaging of clinically asymptomatic patients or on evaluation of terminally sick and morbid cases of fulminant hepatic failure [6]. Abdominal pain and constitutional symptoms are the usual clinical symptoms [11]. Weight loss, night sweats and fever which constitute the B symptoms of lymphoma may be seen in 40-85% patients of PHL [13]. Liver enlargement is present in almost all patients [13]. More than half the patients have significant weight loss, as seen in our patient too [11]. However jaundice which was the presenting symptom in our patient is only seen in 4% [10]. Patients with PHL have deranged liver function tests, as also seen in our patient. Most commonly LDH and alkaline phosphatise levels are affected [11]. The AFP and CEA levels are usually normal and are indicative of a hepatocellular or metastatic cancer respectively. This was also seen in our patient and prompted a biopsy for evaluation of tumor histopathology. The most common imaging manifestation (seen in approximately 50% of patients) in the liver is that of multiple lesions, as seen in our patient [11]. Gold standard of diagnosis is histopathology with add-on immunohistochemical typing and flow-cytometric studies for confirmation. Features on MRI include good soft tissue resolution of the mass which enables better delineation of tumor tissue versus normal liver parenchymal involvement. On T1-weighted signal, these tumors are hypointense to isointense compared to the liver parenchyma and are mild to moderately hyperintense on T2-weighted images. Diffusion restriction is present on echo planar imaging. Post contrast administration, there is mild enhancement on the arterial phase with delayed phase peripheral hypointensity and contrast pooling within the center of the mass. This feature has also been used to differentiate it from hepatocyte origin tumors such as hepatocellular cancer and was also present in our patient. It has also been shown that hepato-biliary specific contrast does not show significant enhancement of PHL during dynamic scanning. Our patient showed diffuse soft tissue infiltration of the right lobe and liver hilum; however, its extension beyond the liver contours to the right kidney was delineated due to the superior soft tissue resolution of MRI. This soft tissue extension however is the most unique aspect of this case. As seen in most cases, the histopathology of the above tumor was diffuse large B-cell primary hepatic lymphoma (DLBCL). T-cell PHLs are reported less frequently.

Our report extends the range of clinical manifestations from solitary, multiple or diffuse lesions to a locally infiltrating tumor extending beyond the liver capsule. Recognition of such an entity is important for the sake of both diagnosis and management of PHL presenting as obstructive jaundice with extent beyond the liver. Prognosis remains favorable in appropriately treated cases.

### Conclusion

DLBC PHL of the liver is a rare diagnosis on imaging. This case illustrates the importance of imaging and histopathology correlation in patients presenting with malignant obstructive jaundice. The above case has demonstrated a unique presentation of PHL with biliary, portal vein, perihepatic fat planes involvement as well as contiguous extension of tumor tissue into the right kidney. This report will help clinicians, radiologists, oncologists and pathologists to consider this rare tumor as a differential diagnosis for cholangiocarcinoma as well as a possible cause for malignant obstructive jaundice which can be effectively treated with non-surgical means, offering a favorable prognosis.

## Source(s) of Support

Nil.

## Abbreviations

DLBC NHL: diffuse large cell B-cell non-Hodgkin lymphoma
PHL: primary hepatic lymphoma
HBV: hepatitis B virus
HCV: hepatitis C virus
EBV: Epstein-Barr virus
